# Early EEG for outcome prediction of postanoxic coma: prospective cohort study with cost-minimization analysis

**DOI:** 10.1186/s13054-017-1693-2

**Published:** 2017-05-15

**Authors:** Lotte Sondag, Barry J. Ruijter, Marleen C. Tjepkema-Cloostermans, Albertus Beishuizen, Frank H. Bosch, Janine A. van Til, Michel J. A. M. van Putten, Jeannette Hofmeijer

**Affiliations:** 1 Department of Neurology, Rijnsate Hospital, Wagnerlaan 55, 6815AD Arnhem, The Netherlands; 2 University of Twente, clinical neurophysiology, Enschede, The Netherlands; 3 Medisch Spectrum Twente, neurology and clinical neurophysiology, Enschede, The Netherlands; 4 Medisch Spectrum Twente, intensive care, Enschede, The Netherlands; 5 Rijnstate Hospital, intensive care, Arnhem, The Netherlands; 6 University of Twente, department of Health Technology and Services Research, Enschede, The Netherlands

**Keywords:** Cardiac arrest, Brain anoxia, Coma, Electroencephalogram, Predictive value of tests, Cost minimization analysis

## Abstract

**Background:**

We recently showed that electroencephalography (EEG) patterns within the first 24 hours robustly contribute to multimodal prediction of poor or good neurological outcome of comatose patients after cardiac arrest. Here, we confirm these results and present a cost-minimization analysis. Early prognosis contributes to communication between doctors and family, and may prevent inappropriate treatment.

**Methods:**

A prospective cohort study including 430 subsequent comatose patients after cardiac arrest was conducted at intensive care units of two teaching hospitals. Continuous EEG was started within 12 hours after cardiac arrest and continued up to 3 days. EEG patterns were visually classified as unfavorable (isoelectric, low-voltage, or burst suppression with identical bursts) or favorable (continuous patterns) at 12 and 24 hours after cardiac arrest. Outcome at 6 months was classified as good (cerebral performance category (CPC) 1 or 2) or poor (CPC 3, 4, or 5). Predictive values of EEG measures and cost-consequences from a hospital perspective were investigated, assuming EEG-based decision- making about withdrawal of life-sustaining treatment in the case of a poor predicted outcome.

**Results:**

Poor outcome occurred in 197 patients (51% of those included in the analyses). Unfavorable EEG patterns at 24 hours predicted a poor outcome with specificity of 100% (95% CI 98–100%) and sensitivity of 29% (95% CI 22–36%). Favorable patterns at 12 hours predicted good outcome with specificity of 88% (95% CI 81–93%) and sensitivity of 51% (95% CI 42–60%). Treatment withdrawal based on an unfavorable EEG pattern at 24 hours resulted in a reduced mean ICU length of stay without increased mortality in the long term. This gave small cost reductions, depending on the timing of withdrawal.

**Conclusions:**

Early EEG contributes to reliable prediction of good or poor outcome of postanoxic coma and may lead to reduced length of ICU stay. In turn, this may bring small cost reductions.

## Background

Anoxic brain damage after cardiac arrest is one of the most common causes of coma worldwide. In Europe alone, approximately 176,000 patients are admitted, yearly. Of all comatose patients after cardiac arrest surviving to hospital admission, 40–66% never regain consciousness as a result of severe postanoxic encephalopathy [1, 2]. With current conventional measures, only 10–20% of patients with a poor outcome can be detected reliably [3]. This indicates that the prognosis remains uncertain in the majority of patients. Consequently, these are treated on intensive care units (ICUs) for up to weeks or months. The burden of lasting uncertainty on families is large and the costs are high.

Early identification of those without potential for recovery of brain functioning contributes to good communication between doctors and families and can prevent inappropriate continuation of intensive care treatment. We established that evolving electroencephalography (EEG) patterns within the first 24 hours after cardiac arrest are a robust contributor to prediction of either good or poor outcome [4]. Lasting isoelectric or low-voltage patterns at 24 hours after arrest were invariably associated with a poor outcome, whereas continuous rhythms within 12 hours were identified as the first reliable predictor of a good outcome. In a prediction model, EEG parameters were complementary to classical outcome predictors (absence of pupillary light responses or absence of somatosensory evoked potentials (SSEPs)): in approximately 10% of patients with a poor outcome all three markers of a poor outcome were present, half had two predictors, and in 40% only one was observed. Based on unfavorable EEG together with classical predictors, 50% of patients with a poor outcome could be identified without false positives. Additionally, 50% of patients with a good outcome could be identified.

In the present prospective study, we confirm predictive values of raw values of early EEG activity for prediction of either poor or good outcome in the largest published cohort of comatose patients on continuous EEG monitoring after cardiac arrest. Additionally, we present a preliminary analysis of the cost-consequences of EEG-based discontinuation of intensive care treatment.

## Methods

### Design

A prospective cohort study was conducted in the ICUs of two teaching hospitals in the Netherlands. In Medisch Spectrum Twente (MST) (Enschede), patients were included from May 2010 to December 2015. In Rijnstate Hospital (Arnhem), patients were included from June 2012 to November 2015. Some of the EEG results from the first 277 patients were reported previously [4].

### Patients

Consecutive, adult patients who were comatose after cardiac arrest (Glasgow Coma Scale score ≤8) and were admitted to the ICU were included. Exclusion criteria were acute stroke, severe traumatic brain injury, progressive neurodegenerative disease, or pre-existing dependency in daily living (cerebral performance category (CPC) 3 or 4)). EEG monitoring was not started between 8 p.m. and 8 a.m. for practical reasons.

### Treatment

Patients were treated according to standard protocols for patients who are comatose after cardiac arrest, including targeted temperature management at 33 °C or 36 °C. Details have been described previously [4].

### Decisions on withdrawal of treatment

Withdrawal of treatment was considered at 72 hours or later, during normothermia, and off sedation. Decisions on treatment withdrawal were based on international guidelines including cases of incomplete return of brainstem reflexes, treatment-resistant myoclonus, and bilateral absence of evoked median nerve SSEPs [5]. Decisions on treatment withdrawal were sporadically taken between 48 and 72 hours in the case of negative SSEP responses. EEG within 72 hours was not taken into account.

### EEG recordings and analyses

EEG recordings with 21 silver–silverchloride cup electrodes were started as soon as possible after arrival at the ICU and continued for at least 3 days, or until discharge from the ICU [4]. For detection of epileptiform discharges, the EEG was followed three-hourly by the neurologist, clinical neurophysiologist, or clinical neurophysiology laboratory technician, but not by the treating intensive care team. In case of seizures warranting treatment, the intensive care team was informed. All other EEG analyses were performed offline after the recordings, blinded to the point in time of the epoch, the patient’s clinical status during the recording, and the outcome. Epochs of 5 minutes were randomly selected by a computer algorithm at 12 and 24 hours after cardiac arrest, as described previously [6]. Data were independently visually analyzed and classified by two reviewers (BR, MT-C, MvP, or JH). The reviewer was allowed to skip an epoch in the case of abundant artifacts. Upon disagreement, consensus was determined by consultation of a third reviewer. Epochs were classified as isoelectric, low-voltage (<20 μV), epileptiform (including evolving seizures and generalized periodic discharges (GPDs)), burst-suppression, diffusely slowed, or normal. “Diffusely slowed” was a continuous pattern with a dominant frequency <8 Hz. “Normal EEG” was a continuous pattern with a dominant frequency ≥8 Hz. Reactivity and anterior-posterior differentiation were not included in the definition of a normal pattern. “Burst-suppression” was defined as increases in amplitude with a duration of at least 500 ms with three or more phases (bursts), alternated with intervals of ≥1 s with activity <10 μV (suppressions). Burst-suppression patterns were subdivided into patterns with and without identical bursts [7]. EEG data were subsequently subdivided into unfavorable (isoelectric, low-voltage, or burst-suppression with identical bursts), intermediate (evolving seizures, GPDs, or burst-suppression without identical bursts), and favorable patterns (continuous patterns, either diffusely slowed, or normal).

### Outcome

The primary outcome measure was the CPC at 6 months after cardiac arrest, dichotomized as good (CPC of 1 or 2) or poor (CPC of 3, 4, or 5). CPC scores were obtained by telephone follow up at 6 months, based on a Dutch translation of the EuroQol-6D questionnaire, blinded for EEG patterns.

### Economic evaluation

Cost-consequences were investigated from a healthcare provider (in-hospital) perspective using a decision analytical approach, incorporating “diagnostic”’ probabilities from the cohort study and costs of current care [8, 9]. We calculated the costs of the current hospital admission (days in ICU, days in general ward (GW), SSEP and, for pathway 2 and 3, costs of an EEG). Costs for readmission, admission to nursing homes, chronic cardiac care, and rehabilitation centers were not taken into account. We constructed a decision tree and estimated costs of care for the following pathways:

- Pathway 1: current care.
- Pathway 2: theoretical withdrawal of life-sustaining treatment at 24 hours after cardiac arrest in patients with EEG patterns that invariably predicted poor outcome at 24 hours.
- Pathway 3: theoretical withdrawal of life-sustaining treatment at 72 hours after cardiac arrest in patients with EEG patterns that invariably predicted poor outcome at 24 hours.

**Fig. 1:**
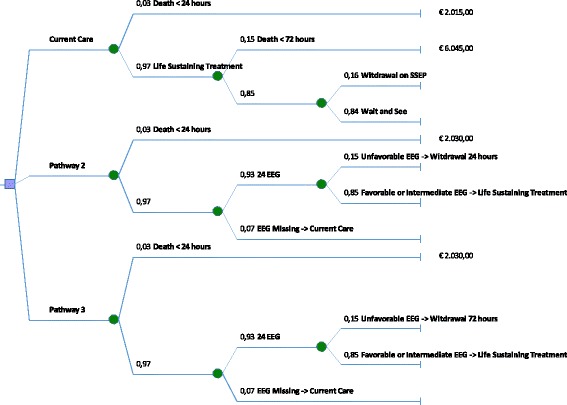
Decision tree. Pathway 1 represents current care of all patients in this study. Pathway 2 and 3 show the estimated flow of patients assuming withdrawal of life-sustaining treatment 24 or 72 hours after cardiac arrest, respectively, in the case of an unfavorable electroencephalography (*EEG*) pattern at 24 hours. Patients in pathway 2 and 3 with a favorable, intermediate, or un-assessable EEG pattern at 24 hours followed the pathway of current care. The *fractions* indicate the numbers of patients that were allocated to a specific branch of the tree. *Prices* indicate estimated costs per patient. In scenario 1 (*current care*) these costs consist of the costs of stay in the intensive care unit and general ward, plus the costs of a contingent performed somatosensory evoked potential (SSEP). In scenario 2 and 3 (*pathway 2* and *pathway 3*), the costs consist of the costs of stay in the intensive care unit and general ward plus the costs of the performed EEG and the costs of a contingent performed SSEP

The pathway of current care was followed for patients in pathway 2 and 3 with favorable, intermediate, or un-assessable EEG patterns at 24 hours. Each branch of the tree represents a day, i.e., branch 1 represents 1 day in ICU, the next branch represents 2 days etcetera. We used probabilities at each branch, calculated by the number of patients that are in that branch related to the total number of patients on the day before (Fig. 1). We calculated costs of care per patient by multiplying the probabilities with the costs for that pathway. Similar, costs of SSEPs were based on the probability that a patient underwent an SSEP.

For pathway 2 and 3, cost reductions for patients with an unfavorable EEG pattern at 24 hours were calculated, given theoretical withdrawal of life-sustaining treatment at 24 or 72 hours. It is assumed that EEG patterns are collected at 24 hours in all patients who are still alive. In patients in pathway 2 and 3 with an unfavorable EEG at 24 hours, theoretical treatment withdrawal was based solely on the EEG pattern at 24 hours. For these patients, we did not calculate the costs for an SSEP. For all other patients in pathway 2 and 3 (i.e., those with favorable or intermediate EEG patterns) costs for SSEPs were taken into account, since in this subgroup the prognosis was unclear from the EEG. Costs of other diagnostic or therapeutic steps were assumed to be largely outweighed by the costs of ICU admission and were not individually collected. The average time between theoretical withdrawal of life-sustaining treatment and death was estimated based on the actual interval according to the collected data: we assumed that this interval did not change per patient with earlier, EEG-based withdrawal. Reference prices are: one day in the ICU €2015, one day in a general ward (GW) €443, SSEP €149, EEG €21 [6].

### Statistical analysis

Analyses were performed using SPSS 22. Baseline characteristics are presented in a descriptive way. Groups of patients with good and poor neurologic outcome were compared. Categorical variables were analyzed using Pearson’s chi-squared test (if no subgroup had an expected count <5) or Fisher’s exact test. Continuous variables were analyzed using the independent samples *t* test or Mann–Whitney *U* test, after checking for normality. Sensitivity, specificity, positive predictive value (PPV), and negative predictive value (NPV) of (groups of) EEG patterns for prediction of outcome after 12 or 24 hours were calculated, including the corresponding 95% confidence interval (CI). Interobserver agreement for visual EEG categorization was analyzed by calculating Cohen’s kappa values. *P* values below 0.05 were considered statistically significant.

## Results

**Fig. 2:**
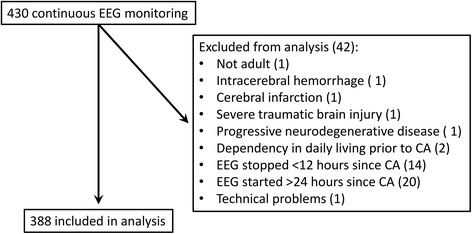
Flow of patients through this study. *CA* cardiac arrest, *EEG* electroencephalography

Continuous EEG monitoring was started in 430 comatose patients and 42 patients were excluded from further analysis (Fig. 2); 388 patients were included in the analysis, 238 from MST and 150 from Rijnstate. Four of the patients included were lost to follow up. In 10 others, the outcome at 6 months was unknown and the CPC score at 3 months was used. There were 197 patients (51%) who had poor outcome, of whom 177 died. Patient characteristics and differences between groups of patients with good and poor outcome are presented in Table 1. The differences were as expected: patients with a good outcome were younger and more often had a cardiac arrest due to cardiac problems, mostly ventricular fibrillation. An SSEP was less frequently performed in the group with a good outcome.

**Table 1:** Patient characteristics and differences between groups of patients with good or poor outcome

Characteristic	Good outcome *N* = 187	Poor outcome *N* = 197	*P* value
Female gender	46 (25%)	55 (28%)	0.6
Mean age (±SD)	60 ± 12	67 ± 12	<0.0001
OHCA	173 (93%)	172 (87%)	0.2
Cardiac etiology	165 (88%)	138 (70%)	<0.0001
VF rhythm	169 (90%)	114 (58%)	<0.0001
Patients treated with propofol	174 (93%)	171 (87%)	0.05
Mean propofol dose (mg/kg/h ± SD)	2.9 ± 1.6	3.2 ± 9.0	0.6
Patients treated with midazolam	74 (40%)	79 (40%)	1.0
Mean midazolam dose (μg/kg/h ± SD)	93 ± 65	112 ± 85	0.2
Patients treated with morphine	70 (37%)	61 (31%)	0.7
Mean morphine dose (μg/kg/h ± SD)	18 ± 13	21 ± 16	0.3
SSEP performed	39 (21%)	139 (71%)	<0.0001
Bilaterally absent SSEP at 72 hours	7 (4%)	65 (33%)	<0.0001
Unfavorable EEG at 24 hours	0/178 (0%)^a^	52/179 (29%)^a^	<0.0001
Favorable EEG at 12 hours	63/123 (51%)^b^	15/125 (12%)^b^	<0.0001

*EEG* electroencephalography, *Favorable EEG* continuous pattern, diffusely slowed or normal, *OHCA* out of hospital cardiac arrest, *SSEP* somatosensory evoked potential, *SD* standard deviation, *Unfavorable EEG* isoelectric, low-voltage, or burst-suppression with identical bursts, *VF* ventricular fibrillation. ^a^Patients who died within 24 hours of cardiac arrest or who had abundant artifacts on EEG are not included here. ^b^Patients in whom EEG was started later than 12 hours after cardiacarrest or who had abundant artifacts on EEG are not included here

Continuous EEG was started at a median of 6.8 hours (interquartile range 22.6) after cardiac arrest. Epochs at 12 hours were missing in 139 patients, because 127 patients had recordings started later than 12 hours, and in 12 patients the computer algorithm did not select an epoch because there were too many artifacts. Epochs at 24 hours were missing in 28 patients, because 11 patients had died within 24 hours, and in 13 patients the algorithm did not select an epoch because there were too many artifacts; in 4 patients the reason was not reported. Epochs could not be visually interpreted in 7 patients at 12 hours and in 4 patients at 24 hours because of artifacts. Patients with and without sporadic missing EEG data did not differ in terms of demographic parameters, clinical parameters, EEG parameters, or outcome. A median nerve SSEP was performed in 178 patients. Withdrawal of life-sustaining treatment was based on bilateral absent SSEP responses in 52 patients. In the remaining patients, eventual withdrawal of treatment was based on lack of neurologic improvement, other organ failure, or a combination of these.

### EEG prediction of neurological outcome

An unfavorable EEG pattern (isoelectric, low voltage, or burst-suppression with identical bursts) was present in 52 patients at 24 hours, and this was unequivocally associated with a poor outcome (1 patient had a CPC score of 3 and 51 patients died). In the 79 patients with a favorable EEG pattern (normal or diffusely slowed) at 12 hours after cardiac arrest, 63 (80%) had a good neurological outcome (Table 1). Sensitivity, specificity, and predictive values of EEG categories for the prediction of good or poor outcome are presented in Table 2.

**Table 2:** Predictive values of EEG patterns

Favorable or unfavorable pattern	Time since cardiac arrest	Predicted outcome	Specificity (95% CI)	Sensitivity (95% CI)	PPV (95% CI)	NPV (95% CI)
Favorable EEG pattern	12 h	Good	88% (81‒93)	51% (42‒60)	81% (70‒88)	65% (57‒72)
Unfavorable EEG pattern	24 h	Poor	100% (98‒100)	29% (22‒-36)	100% (93‒100)	58% (52‒64)

*EEG* electroencephalography, *Favorable EEG* continuous pattern, diffusely slowed or normal, *Unfavorable EEG* isoelectric, low-voltage, or burst-suppression with identical bursts, *PPV* positive predictive value indicating the probability that patients with a positive test result truly have the predicted outcome (a PPV of 100% therefore indicates 0% false positives), *NPV* negative predictive value indicating the probability that patients with a negative test result truly do not have the predicted outcome, *CI* confidence interval

### Interobserver agreement

The inter observer agreement for determining a favorable EEG at 12 hours was 0.65 (95% CI 0.55–0.76). For determination of an unfavorable EEG at 24 hours, this was 0.64 (95% CI 0.56–0.72). For determining a favorable EEG, disagreements related to the distinction between a waxing and waning burst suppression pattern and a continuous pattern. For determining an unfavorable EEG, most disagreements related to the distinction between low voltage and burst suppression (when sporadic activity of more than 20 μV was observed), and the distinction between burst suppression with and without identical burst.

### Consequences for survival and costs of EEG-based withdrawal of life-sustaining treatment

Among patients receiving current care (pathway 1), 177 patients had died 6 months after cardiac arrest, 161 in the ICU, 6 in other hospital departments (after discharge from the ICU), and 2 after discharge from the hospital; where the remaining 8 patients had died was unknown. Of 161 patients who died in the ICU, 10 died within 24 hours. Twenty patients with a poor neurological outcome were alive at 6 months after cardiac arrest and had a CPC score of 3. The mean (standard deviation (SD)) days in the ICU and in a GW, was 8.2 (10.8) and 9.6 (12.7), respectively.

**Fig. 3:**
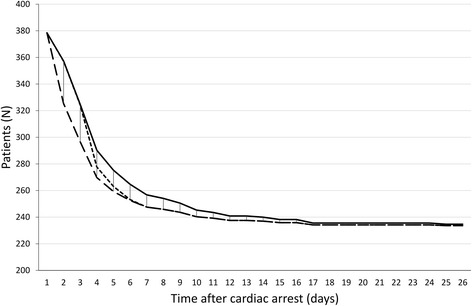
Survival curves. The *solid line* represents the actual survival curve for the cohort, the *lower broken line* represents the estimated survival assuming withdrawal of life-sustaining treatment at 24 hours in patients with an unfavorable electroencephalography (EEG) pattern at 24 hours after cardiac arrest, and the *upper broken line* the estimated survival assuming withdrawal of life-sustaining treatment at 72 hours in patients with an unfavorable EEG pattern at 24 hours after cardiac arrest. These curves indicate a decrease in survival in the first 2 weeks, but no increased mortality in the long term

Among patients who died, the mean (SD) time between cardiac arrest and withdrawal of life-sustaining treatment was 4.6 (2.9) days. Of 161 patients who died in the ICU, 51 had unfavorable EEG patterns at 24 hours. Of twenty patients with a CPC of 3 at 6 months, one had an unfavorable EEG pattern at 24 hours. If, based on the EEG patterns, decisions on withdrawal of life-sustaining treatment had been taken at 24 hours (pathway 2) or 72 hours (pathway 3), the survival curves would obviously have been different. This is illustrated in Fig. 3, showing that these two scenarios would lead to reduced survival in the first week. However, given the high specificity of unfavorable EEG patterns for prediction of a poor outcome, mortality in the long term would remain unchanged. The reduction in mean days in the ICU and GW among patients with an unfavorable EEG at 24 hours would be 2.9 for decision of withdrawal at 24 hours and 1.1 for decision of withdrawal at 72 hours. If treatment withdrawal was decided after 24 hours based on an unfavorable EEG pattern, this would result in a cost reduction of €334 per patient and €126.591 for the total group of 388 patients. If treatment withdrawal was decided after 72 hours based on an unfavorable EEG pattern at 24 hours, this would not lead to cost reduction (Table 3).

**Table 3:** Admission days of patients with unfavorable EEG patterns at 24 hours and hospital costs for pathways 1, 2, and 3

Number of patients, number of days, and costs	Pathway 1	Pathway 2	Pathway 3
Patients with unfavorable EEG at 24 hours (*n* = 52)			
Alive at 6 months	1	1	1
Deceased at 6 months	51	51	51
Mean days ICU (SD)	4.1 (1.8)	1.2 (0.7)	3.0 (0.8)
Mean days GW (SD)	0.6 (4.6)	0.6 (4.6)	0.6 (4.6)
All patients (*n* = 388)			
Costs per patient	€19.285,-	€18.951,-	€19.461,-
Total costs for cohort	€7.308.832,-	€7.182.241,-	€7.375.681,-

*EEG* electroencephalography, *ICU* intensive care unit, *GW* general ward, *SD* standard deviation

## Discussion

An unfavorable EEG pattern at 24 hours predicted a poor outcome without false positives. In the case of a favorable EEG pattern within 12 hours, the probability of a good neurological recovery was strong and survival depended on failure of organs other than the brain. These findings are in agreement with our previous work [4, 10, 11] and that of others [12]. The current PPV of a favorable EEG pattern was slightly lower than in our previous reports, because of slightly higher mortality from non-neurological causes. With a multimodal analysis, we recently showed that the EEG is complementary to conventional outcome predictors [4].

Cost minimization of an EEG-based policy, using an unfavorable EEG pattern at 24 hours as a rule to withdraw life-sustaining treatment, was small and depended on the timing of decision-making. This is explained by the fact that continuous EEG recordings had to be performed in all 388 patients, to obtain a reduction in ICU stay for 51 patients. The small reduction is probably related to the liberal Dutch practice of prompt discontinuation of treatment in most patients with a negative SSEP. The reduction is probably an underestimation, as we assumed that for each patient the number of days remaining after withdrawal of life support was the same with early as compared with late withdrawal. However, withdrawal of life-sustaining treatment within the first 3 days would probably have led to faster death than later withdrawal. Furthermore, in this analysis, we only took in-hospital costs into account. From a societal perspective, cost reductions are probably higher, resulting from a reduction in chronic admissions to nursing homes.

Apart from financial profits, obvious qualitative benefits of early reliable prognosis include reduction in the uncertainty when providing information to family members about the severity of brain damage and the patient’s subsequent prognosis. This also holds for the medical team, as the impact of both withdrawal and continuation of intensive care is large.

Whereas most studies assume that the reliability of EEG in designating the severity of encephalopathy increases with time [5], we repeatedly observed the highest predictive values within 24 hours of cardiac arrest, even with the use of hypothermia and sedative medication [4, 6, 13]. Apparently, in postanoxic encephalopathy, improvement of brain activity up to a minimum level within 24 hours is essential. Beyond 24 hours, there is evolution toward less specific patterns, even in patients who eventually have a poor outcome [4]. Whether or not these patterns still include features allowing prognosis remains to be analyzed [6].

We stress that predictive values of the EEG are high, despite the use of hypothermia and sedative medication. Isoelectric, low-voltage, or burst-suppression with identical burst patterns cannot be solely induced by hypothermia, propofol, or midazolam. Cooling the brain to 32 °C influences the EEG only slightly and propofol-induced EEG changes are well-known. At the dosages that were used in our patients, the EEG mostly remains continuous, with anteriorization of the “alpha” rhythm [14]. If burst-suppression is induced by propofol or midazolam, bursts are heterogeneous and appear and disappear gradually [15], whereas our identical burst-suppression patterns are all characterized by flat inter-burst intervals and abrupt transitions between bursts and suppressions [7]. Still, with sedative medication in substantially higher dosages than described in Table 1, our results should be interpreted with caution.

A potential limitation of all studies investigating diagnostic accuracy is the self-fulfilling prophecy. This characterizes almost all studies on this topic [16–19]. Guidelines for treatment continuation were strictly followed, and these do not include the EEG during the first 72 hours. However, attending physicians were not blinded to the EEG results, to allow treatment of epileptic phenomena. Second, visual analysis of EEG data is subject to interrater variability. Interobserver agreement was 0.64, which is higher than the values of 0.20–0.65 reported for the SSEP [18, 19]. Third, we only used a rough measure for good or poor outcome at 6 months. We did not include a detailed calculation of costs for every diagnostic and therapeutic step and we did not calculate quality-adjusted life years. Fourth, our study does not resolve ethical dilemmas about the translation of the predictive values in this study at a population level to treatment decisions in individual patients.

## Conclusions

We confirm the value of early EEG measurements for reliable prediction of good or poor outcome of comatose patients after cardiac arrest. EEG-based decisions on treatment (dis)continuation may lead to a reduction in the ICU length of stay, without increased mortality in the long term. In turn, this may bring cost reductions, depending on the timing of withdrawal of life-sustaining treatment.

## Abbreviations

CI: Confidence interval
CPC: Cerebral performance category
GPD: Generalized periodic discharges
EEG: Electroencephalography
GW: General ward
ICU: Intensive care unit
MST: Medisch Spectrum Twente hospital
NNP: Negative predictive value
PPV: Positive predictive value
SD: Standard deviation
SSEP: Somatosensory evoked potential

## References

[1] Zandbergen EG, de Haan RJ, Stoutenbeek CP (1998). Systematic review of early prediction of poor outcome in anoxic-ischaemic coma. Lancet..

[2] Bernard SA, Gray TW, Buist MD (2002). Treatment of comatose survivors of out-of-hospital cardiac arrest with induced hypothermia. N Engl J Med..

[3] Sandroni C, Cariou A, Cavallaro F (2014). Prognostication in comatose survivors of cardiac arrest: an advisory statement from the European Resuscitation Council and the European Society of Intensive Care Medicine. Intensive Care Med..

[4] Hofmeijer J, Beernink TM, Bosch FH (2015). Early EEG contributes to multimodal outcome prediction of postanoxic coma. Neurology..

[5] Wijdicks EF, Hijdra A, Young GB, Quality Standards Subcommittee of the American Academy of Neurology, et al. Practice parameter: prediction of outcome in comatose survivors after cardiopulmonary resuscitation (an evidence-based review): report of the Quality Standards Subcommittee of the American Academy of Neurology. Neurology. 2006;67:203–10. 10.1212/01.wnl.0000227183.21314.cd 16864809

[6] Tjepkema-Cloostermans MC, van Meulen FB, Meinsma G, van Putten MJ (2013). A Cerebral Recovery Index (CRI) for early prognosis in patients after cardiac arrest. Crit Care..

[7] Hofmeijer J, Tjepkema-Cloostermans MC, van Putten MJ (2014). Burst-suppression with identical bursts: a distinct EEG pattern with poor outcome in postanoxic coma. Clin Neurophysiol..

[8] Zorginstituut Nederland. guideline for economical analyses in health care. 2015. available at: https://www.zorginstituutnederland.nl/pakket/werkwijze + pakketbeheer/beoordeling + geneesmiddelen/economische + evaluatie, accessed 01-07-2016.

[9] Sculpher M, Drummond M, Buxton M (1997). The interative use of economic evaluation as part of the process of health technology assesment. J Health Servs Res Policy..

[10] Tjepkema-Cloostermans MC, Hofmeijer J, Trof RJ (2015). Electroencephalogram predicts outcome in patients with postanoxic coma during mild therapeutic hypothermia. Crit Care Med..

[11] Hofmeijer J, van Putten MJ (2016). EEG in postanoxic coma: Prognostic and diagnostic value. Clin Neurophysiol..

[12] Sivaraju A, Gilmore EJ, Wira CR (2015). Prognostication of post-cardiac arrest coma: early clinical and electroencephalographic predictors of outcome. Intensive Care Med..

[13] Cloostermans MC, van Meulen FB, Eertman CJ (2012). Continuous electroencephalography monitoring for early prediction of neurological outcome in postanoxic patients after cardiac arrest: a prospective cohort study. Crit Care Med..

[14] Hindriks R, van Putten MJAM (2012). Meanfield modeling of propofol-induced changes in spontaneous EEG rhythms. Neuroimage..

[15] Kusters AH, Vijn PC, van den Brom WE, Haberham ZL, Venker-van Haagen AJ, Hellebrekers LJ (1998). EEG-burst-suppression-controlled propofol anesthesia in the dog. Vet Q..

[16] Rossetti AO, Tovar Quiroga DF, Juan E, Novy J, White RD, Ben-Hamouda N, Britton JW, Oddo M, Rabinstein AA. Electroencephalography Predicts Poor and Good Outcomes After Cardiac Arrest: A Two-Center Study. Crit Care Med. 2017. doi:10.1097/CCM.0000000000002337. [Epub ahead of print]. 10.1097/CCM.0000000000002337 28406812

[17] Rossetti AO, Oddo M, Logroscino G, Kaplan PW (2010). Prognostication after cardiac arrest and hypothermia: a prospective study. Ann Neurol..

[18] Oddo M, Rossetti AO (2014). Early multimodal outcome prediction after cardiac arrest in patients treated with hypothermia. Crit Care Med..

[19] Zandbergen EG, Hijdra A, Koelman JH (2006). Prediction of poor outcome within the first 3 days of postanoxic coma. Neurology..

